# Repeated Excessive Exercise Attenuates the Anti-Inflammatory Effects of Exercise in Older Men

**DOI:** 10.3389/fphys.2017.00407

**Published:** 2017-06-22

**Authors:** Ronni E. Sahl, Peter R. Andersen, Katja Gronbaek, Thomas H. Morville, Mads Rosenkilde, Hanne K. Rasmusen, Steen S. Poulsen, Clara Prats, Flemming Dela, Jørn W. Helge

**Affiliations:** ^1^Xlab, Center for Healthy Aging, University of CopenhagenCopenhagen, Denmark; ^2^Department of Biomedical Sciences, University of CopenhagenCopenhagen, Denmark; ^3^Department of Cardiology, Bispebjerg and Frederiksberg Hospitals, University of CopenhagenCopenhagen, Denmark; ^4^Department of Geriatrics, Bispebjerg University Hospital, University of CopenhagenCopenhagen, Denmark

**Keywords:** prolonged exercise, cytokines, skeletal muscle, macrophages, low-grade inflammation

## Abstract

**Introduction/Purpose:** A number of studies have investigated the effect of training with a moderate exercise dose (3–6 h/weekly) on the inflammatory profile in blood, and the data are inconsistent. Cross-sectional studies indicate a positive effect of physical activity level on inflammation levels and risk of metabolic disease. However, it is not clear whether this may be dose dependent and if very prolonged repeated exercise therefore may be beneficial for low-grade inflammation. Based on this we studied how excessive repeated prolonged exercise influenced low-grade inflammation and adipose tissue anti-inflammatory macrophage content in six older male recreationally trained cyclists. Low-grade inflammation and adipose tissue macrophage content were investigated in six older trained men (age: 61 ± 4 years; VO_2peak_: 48 ± 2 mL kg^−1^ min^−1^) following repeated prolonged exercise.

**Methods:** Cycling was performed daily for 14 days covering in total 2,706 km (1,681 miles). Maximal oxygen uptake (VO_2peak_) was measured before and after the cycling. Duration and intensity of the exercise were determined from heart rates sampled during cycling. An adipose tissue biopsy from subcutaneous abdominal fat and a blood sample were obtained at rest in the overnight fasted state before and after the cycling. Anti-inflammatory adipose tissue macrophages (ATM) were immunohistochemically stained in cross sectional sections using a CD163 binding antibody. The ATM and adipocyte sizes were analyzed blindly.

**Results:** The cyclists exercised daily for 10 h and 31 ± 37 min and average intensity was 53 ± 1% of VO_2peak_. Body weight remained unchanged and VO_2peak_ decreased by 6 ± 2% (*P* = 0.04). Plasma inflammatory cytokines, TNFα and IL-18 remained unchanged, as did hsCRP, but plasma IL-6 increased significantly. CD163 macrophage content remained unchanged, as did adipocyte cell size. The HbA1c was not significantly decreased, but there was a trend (*P* < 0.07) toward an increased insulin resistance as estimated by the Quicki Index.

**Conclusion:** The regular prolonged exercise did not influence abdominal adipose tissue inflammation, but the higher plasma IL-6 concentration concurrent with a trend toward higher insulin resistance and decreased VO_2peak_ implies that the excessive amount of exercise probably attenuated the possible potential anti-inflammatory effects of exercise.

## Introduction

Physical inactivity and an unhealthy energy rich diet are often named as primary causes behind the worldwide increase in obesity and type 2 diabetes (WHO, 2016). Therefore, increasing daily physical activity is desirable and is beneficial for the individual as well as for the society. However, it is uncertain if extensive exercise is beneficial. Today there is an increasing number of people that participate in very prolonged exercise; marathons, long distance bike rides, triathlons, and even extreme ultra endurance exercise, yet there are several aspects of very prolonged exercise, where the mechanisms and health effects, positive and negative, are not well described.

Based on cross-sectional data higher physical activity levels are associated with reduced low-grade inflammation and thus lower susceptibility to obesity and type 2 diabetes (Abramson and Vaccarino, 2002; King et al., 2003; Schmidt et al., 2015). The available data from longitudinal studies are inconclusive, with studies showing that training and regular exercise attenuates low-grade inflammation (Larsen et al., 2001; Troseid et al., 2009; Thompson et al., 2010), but other studies, and in addition some very large Randomized Clinical Trials (RCT), have shown no effect on low-grade inflammation (Hammett et al., 2004; Beavers et al., 2013; Cooper et al., 2016). There is evidence that physical activity and weight loss combined attenuates low-grade inflammation (Beavers et al., 2013). Moreover, we previously demonstrated that 15 weeks lifestyle intervention consisting of a hypocaloric diet and physical activity in very obese individuals decreased low-grade inflammation and the density of inflammatory macrophages in abdominal adipose tissue concurrent with an improved insulin sensitivity and metabolic fitness (Bruun et al., 2006; Helge et al., 2011). Interestingly, a RCT in male overweight subjects demonstrated that 12 weeks controlled supervised endurance training with no weight loss did not influence low-grade inflammation, but did induce a higher abdominal adipose tissue anti-inflammatory CD163 macrophage content (Auerbach et al., 2013). The majority of the studies above have applied moderate exercise doses (3–6 h/weekly) and it is not clear whether very prolonged, repeated exercise will be beneficial for the inflammatory profile in blood and adipose tissue.

Based on this, we studied how excessive repeated prolonged exercise influenced low-grade inflammation and adipose tissue anti-inflammatory macrophage content in six older male recreationally trained cyclists. We hypothesized that repeated prolonged exercise without a weight loss would not influence plasma inflammatory markers, but would increase adipose tissue anti-inflammatory macrophage content.

## Materials and methods

### Study design

**Figure 1:**
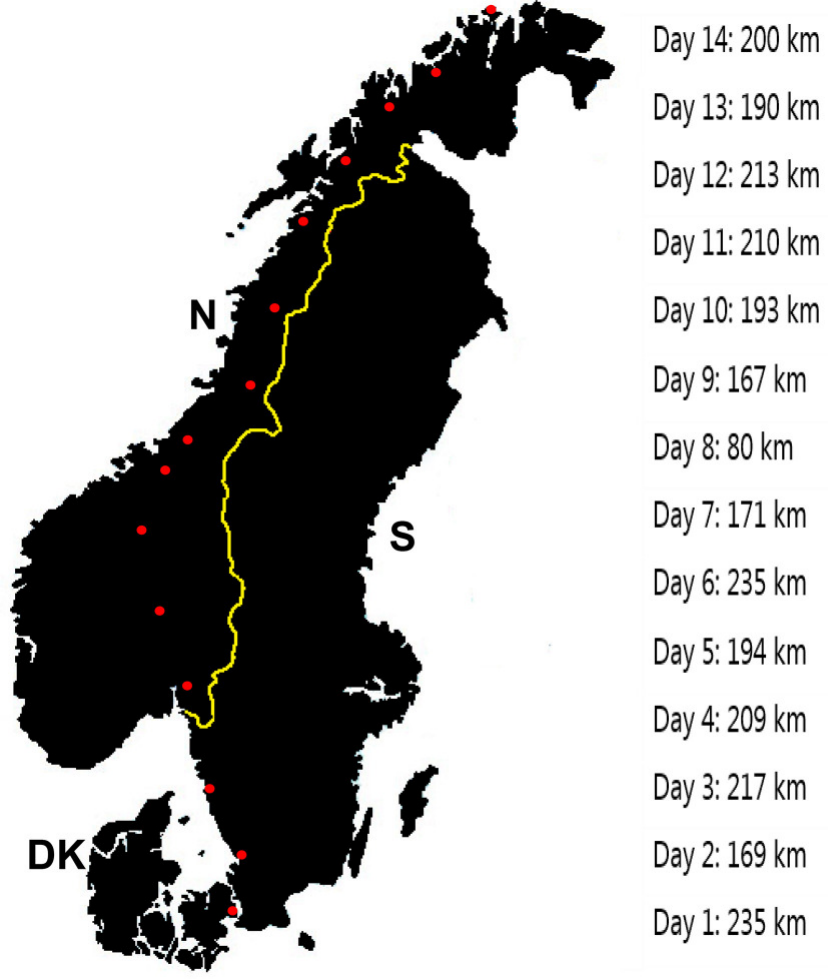
Geographical depiction of the 2,706 km (1,681 miles) route from Copenhagen, Denmark, to North Cape, Norway, via Sweden. The red dots depict departure and arrival points of daily stages throughout the trip. Daily stage distances in kilometers are shown from days 1 to 14. DK, Denmark; N, Norway; S, Sweden. Used by permission (32).

The design of this study has been published in detail elsewhere (Rosenkilde et al., 2015; Morville et al., 2017). In brief, six male recreationally trained cyclists (61 ± 4 years, VO_2peak_ 48 ± 2 mL·kg^−1^·min^−1^) participated in the study. The subjects were all members of a bicycle club and most were active cycling 2–4 times per week in the months prior to departure. On their own accord, to win a bet (not initiated by any of the authors), the subjects organized a 14 days cycle trip with a total distance of 2,706 km (1,681 miles) from the Town Hall Square in Copenhagen, Denmark to North Cape in Norway, as far north as possible on the European continent (Figure 1; Rosenkilde et al., 2015). On this trip, the subjects' own support team handled all the practical logistics, providing transport of extra gear and maintenance of bikes and supplying, cooking, and preparing all food and beverages before, during, and after cycling. Two to five days before leaving and again 28–33 h after arrival at North Cape, the subjects were tested under standardized conditions in the morning after an overnight fast. All subjects were tested using the same equipment and protocols on both occasions. The sampling of blood and adipose tissue were done exactly as before the departure from Copenhagen and efforts were made to do the tests at the same time of day. In the day prior to the test day subjects were asked to refrain from participating in strenuous or prolonged exercise.

### Ethical approval

The subjects were informed about the possible risks and discomfort involved before written consent to participate was obtained. The study was performed according to the Declaration of Helsinki and was approved by the Science Ethical Committee of the Copenhagen Region (H3-2011-008) and registered at clinicaltrials.gov (NCT02353624).

### Experimental conditions

Subjects arrived in the morning overnight fasted and after a 15 min rest a venous blood sample was collected from an antecubital vein. After this, an adipose tissue biopsy was obtained with a Bergström needle with applied suction from subcutaneous adipose tissue 5–7 cm lateral to the umbilicus.

Following this an incremental exercise test applying increments of 40 watts per min until exhaustion in order to determine maximal oxygen uptake. Conventional criteria; a leveling off of VO_2_ despite an increase in power output, a respiratory exchange ratio exceeding 1.15 and achievement of estimated maximal heart rate was used to control the test. The pulmonary values during exercise were measured with an online system (CosMed, Quark b^2^, Rome, Italy).

### Analytical procedures

#### Blood analysis

Blood was transferred into tubes containing 0.3 mol·L^−1^ EDTA (10 μL·ml^−1^ blood) and immediately centrifuged at 4°C for 10 min at 23,000 g. The plasma was stored at –80°C until analysis. Analysis of plasma insulin and glucose has been described previously (Ara et al., 2011). The Quicki index was calculated as previously described (Katz et al., 2000). Blood HbA1c was analyzed on a DCA Vantage Analyzer (Siemens Healthcare, NY, USA). Plasma IL-6 and plasma TNF-α were measured with high-sensitivity ELISA kits from R&D Systems (Minneapolis, MN, USA). The plasma IL-18 was analyzed with HS ELISA (R&D Systems) and CRP using a conventional high-sensitivity assay on a Cobas Hitachi (Roche, Indianapolis, USA). The plasma adiponectin concentration was determined with a human radioassay kit (EMD Millipore. Billerica, MA, USA). The plasma leptin concentration was measured by a high-sensitivity human ELISA kit (Human leptin Immunoassay, R&D systems).

#### Adipose tissue

The description of the adipose tissue fixation and analysis procedure is described in full detail in a prior publication (Auerbach et al., 2013). In brief, the adipose tissue was fixed in 2% Zamboni fixative and embedded in paraffin. At time of analysis the paraffin block was sectioned (5 μm thick) on a microtome (Reichter, Münich, Germany). To determine adipocyte area and the general morphology sections of subcutaneous adipose tissue were stained with Mayer's haematoxylin (S3309, Dako, Copenhagen, Denmark) and 2% eosin (1345, Merck, NJ, USA).

The macrophages were immunostained with a primary antibody against CD163 (diluted 1:200, Visionbiosystems Novocastra, Newcastle, UK) and visualized using Dako's REAL Envision Detection system (K5007, Dako, Copenhagen, Denmark) and 3,3′-diaminobenzidine as a chromogen. Sections with no primary antibody were included as control. The stained sections were scanned by means of a Panoramic slide scanner (Zeiss, Oberkochen, Germany). The quantification was done as described by Cancello et al. (2005) and further qualified by Auerbach et al. (2013). The CD163+ cells and the adipocytes were counted by an observer blinded for slide identity in eight randomly chosen areas (magnified × 20) within each slide, representing an area of 123 ± 7 adipocytes and excluding macrophages located in abundant stroma-vascular areas. The total number of CD163+ macrophages was expressed as a percentage of the total number of adipocytes counted.

The cross-sectional adipocyte area was determined using Adobe PhotoShop CS4 Extended Version 11.0.1 (Adobe Systems Incorporated, San Jose, California, USA). Areas were determined by contrasting differences in color (Figure 1). Background variations due to uneven illumination was compensated with the “high pass filter” function and the “threshold” function was applied to achieve a stronger contrast between adipocyte “content” and adipocyte “wall.” Due to variation in the quality of the fixation, sectioning and staining a lower cut-off value at 10,000 pixels ≈ 633 μm^2^ was used to ensure only adipocytes were quantified. The number of adipocytes quantified per subject was 123 ± 7.

#### Statistical analysis

The statistics were calculated using Sigma Plot ver. 13.0 (Systat Software Inc., San Jose, USA). To assess changes before and after the 14 days of cycling a two-tailed paired *t*-test was used. Results are given as mean ± SEM., if not otherwise stated. In all cases, *P* < 0.05 was used as the level of significance in a two-tailed test.

## Results

### Study characteristics

The characteristics of the cycling trip have been published previously (32). In summary all six subjects completed the full distance of 2,706 km (1,681 miles) in 14 days with an average daily distance of 193 ± 10 km·day^−1^ (range: 167–235 km·day^−1^). Daily exercise time was 10 h and 31 ± 37 min·day^−1^ with an exercise intensity of 53.1 ± 1.1% of VO_2peak_ and approximately 198 ± 58 min·day^−1^ exercise intensity above 60% of VO_2peak_ (Morville et al., 2017). After cycling, maximal oxygen uptake was reduced (3.71 ± 0.17 to 3.49 ± 0.20 L O_2_/min and 48 ± 2 to 45 ± 2 mL O_2_/min/kg, *P* < 0.04) (Morville et al., 2017). The peak workload attained during the VO_2peak_ test was not significantly lowered (*P* = 0.22) from before to after the 14 days 357 ± 31 and 333 ± 24 watt, respectively. As previously reported body weight remained unchanged (Table 1), but we observed a 2.2 ± 0.7 kg loss of fat mass and 2.5 ± 0.6 kg gain of lean mass after 8 days of cycling using an isotope dilution technique (Rosenkilde et al., 2015).

**Table 1:** Anthropometric and body composition data measured at rest overnight fasted before and after 14 days of daily prolonged cycling.

	**Pre**	**Post**	***t*-test**
Age (years)	61.3 ± 8.4	–	–
Height (cm)	178 ± 8	–	–
Weight (kg)	77.4 ± 10.2	77.7 ± 10.6	NS
BMI (kg m^−2^)	24.5 ± 2.2	24.5 ± 2.0	NS
Waist (cm)	83.6 ± 8.1	84.4 ± 5.9	NS

*The values are mean ± SEM*.

Plasma glucose concentrations at rest remained unchanged and there was a trend (*P* = 0.06) toward higher plasma insulin after cycling (Table 2; Morville et al., 2017). The glycosylated hemoglobin was not significantly increased, probably due to the limited time of the experiment, but there was a trend (*P* < 0.07) toward an increase in the quantitative insulin sensitivity check index (QUICKI) (Table 2). Plasma hsCRP, TNFα, and IL-18 were not changed after the 14 days (Table 2), but there was a significantly higher plasma IL-6 concentration (Table 2). The plasma leptin and adiponectin concentrations at rest remained unchanged (Table 2; previously published Rosenkilde et al., 2015; Morville et al., 2017).

**Table 2:** Plasma glucose, insulin and Quicki-index as well as plasma cytokines, hsCRP and adiponectin and leptin measured at rest overnight fasted before and after 14 days of daily prolonged cycling in 6 older males.

	**Pre**	**Post**	***t*-test**
Plasma glucose (mmol L^−1^)	5.9 ± 0.2	6.0 ± 0.3	NS
Plasma Insulin (pmol L^−1^)	16 ± 2	33 ± 2	(*P* < 0.07)
HbA1c (mmol L^−1^)	5.9 ± 0.5	6.4 ± 0.4	NS
Quicki index	0.42 ± 0.01	0.38 ± 0.01	(*P* < 0.07)
Hs CRP (mg dl^−1^)	0.19 ± 0.08	0.17 ± 0.05	NS
Plasma IL-6 (pg ml^−1^)	0.7 ± 0.1	1.1 ± 0.1	(*P* < 0.05)
Plasma IL-18 (pg ml^−1^)	310 ± 44	321 ± 39	NS
Plasma TNFα (pg ml^−1^)	2.1 ± 0.8	2.2 ± 0.8	NS
Plasma leptin (pg ml^−1^)	1,257 ± 201	1,357 ± 177	NS
Plasma Adiponectin (μg ml^−1^)	27.6 ± 1.6	43.5 ± 13.1	NS

*The values are mean ± SD. hsCRP, C-reactive protein; IL-6, interleukin 6; IL-18, Interleukin 18; TNFα, Tumor Necrosis Factor α. The values are mean ± SEM. Plasma insulin, glucose, adiponectin, and leptin have been previously published (Rosenkilde et al., 2015; Morville et al., 2017)*.

**Figure 2:**
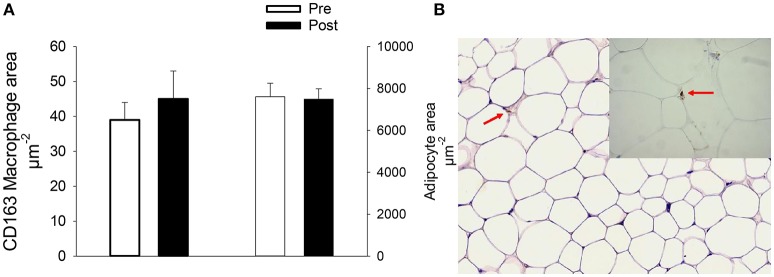
CD163 macrophage content and adipocyte cell size in abdominal adipose tissue **(A)** and a representative picture of CD163 staining (Red arrows) in adipose tissue **(B)** in older male recreationally trained cyclist before and after 14 days repeated very prolonged exercise. The values are mean ± SEM.

The abdominal adipose tissue content of CD163 macrophages were not influenced by the prolonged cycling and likewise the abdominal adipocyte size were also unchanged after the intervention (Figure 2).

## Discussion

In the present study, the hypothesis was refuted, as the major finding was a slightly increased low-grade inflammation and an unchanged anti-inflammatory macrophage content in abdominal adipose tissue after 14 days of excessive exercise.

In line with prior studies we observed no exercise induced changes in plasma hsCRP, TNFα, and IL-18 (Auerbach et al., 2013), but we did see a modest increase in plasma IL-6 concentration and thus a slightly higher low-grade inflammation. Interestingly, there was a lower fat mass after the intervention (Rosenkilde et al., 2015), albeit body weight was unchanged, and if anything this should lead to lowered plasma inflammatory cytokine concentrations (Bruun et al., 2006, 2007; Beavers et al., 2013). There is evidence that cytokine release is positively correlated to adipocyte size (Coppack, 2001) and therefore the unchanged adipocyte cell size, as observed in our study, is consistent with the unchanged inflammatory cytokines, but obviously not the change in plasma IL-6. The plasma leptin and adiponectin concentrations were not changed (data previously reported Rosenkilde et al., 2015; Morville et al., 2017), and this is in line with the unchanged adipocyte cell size reported here. Although we did observe a small decrease in fat mass and a trend toward a minor increase in insulin resistance, as evaluated by the Quicki Index, this did not translate into decreased plasma leptin and adiponectin, probably due to both the short duration of our intervention and the low number of subjects. The mechanism behind the insulin resistance is not readily available as we previously observed both an increased muscle GLUT4 and HKII expression as well as unchanged muscle glycogen, triacylglycerol, and ceramide content (Morville et al., 2017). An increased plasma IL-6 concentration, albeit of a much larger magnitude, has previously been linked to increased lipolysis and fat oxidation at rest (Mathur and Pedersen, 2008), but clearly here, is in contrast to the very marked decrease in maximal fat oxidation and plasma FA concentration observed previously (Morville et al., 2017). Somewhat surprising peak oxygen uptake was reduced by 6% and citrate synthase in muscle, a mitochondrial marker, remained unchanged after the 14 days excessive exercise (Morville et al., 2017), which may indicate that the older males participating in this study were possibly at or just over the maximal tolerable sustainable repeated daily exercise load. It is thus possible that we could not confirm our hypothesis that low-grade inflammation would remain unchanged due to an “overload” of exercise. The mechanisms behind the anti-inflammatory effect of exercise are not fully elucidated in the literature, but includes a reduction in visceral fat, a reduction in expression of toll-like receptor on macrophages and monocytes, production of anti-inflammatory molecules from muscle and leukocytes and a phenotypic switching of macrophages toward the M2 anti-inflammatory state in adipose tissue (Flynn and McFarlin, 2006; Astrom et al., 2010; Goh et al., 2016).

The second part of our hypothesis was also refuted as we could not detect an increase in abdominal adipose tissue CD163 anti-inflammatory macrophage area and thus were not able to confirm Auerbach and colleagues findings of an increased abdominal adipose tissue CD163 macrophage content after 12 weeks endurance training in young overweight males (Auerbach et al., 2013). The available data in man is very limited, but Leggate and colleagues found a decrease in plasma IL-6 and a trend toward a decrease in adiponectin in subcutaneous adipose tissue after 2 weeks high intensity intermittent training in young overweight and obese males implying that even shorter training may also mediate changes in adipose tissue (Leggate et al., 2012). In mice there is evidence, but not conclusive, that training will lead to a switch in macrophage phenotype from M1 (pro-inflammatory) toward M2 [anti-inflammatory (CD163)] (recently reviewed in Kawanishi et al., 2010; Oliveira et al., 2013; Linden et al., 2014; Goh et al., 2016). However, further studies in man are needed to elucidate the anti-inflammatory effects of very prolonged repeated exercise on adipose tissue including the different adipose tissue depots and the mechanisms that control and regulate this.

A limitation in this study was that we due to logistic reasons were not able to obtain blood and tissue samples during the 14 days and therefore the potential changes during the study cannot be distinguished.

In summary, regular prolonged exercise did not influence abdominal adipose tissue anti-inflammatory macrophage content, but the slightly increased low-grade inflammation, higher plasma IL-6 concentration, implies that the excessive amount of exercise in these older trained men probably attenuated the potential anti-inflammatory effects of exercise.

## Author contributions

The study was performed at Xlab, Center for Healthy Aging, Department of Biomedical Sciences, Faculty of Health and Medical Sciences, University of Copenhagen, Denmark. MR, JH conceived and designed the study. All authors collected samples, performed analyses and/or took part in data interpretation. RS and JH wrote the first draft of the manuscript, and all authors took part in critical revision for intellectual content of the manuscript. The final manuscript was presented to and approved by all authors, who agreed to be liable for all aspects of the study. All those who were eligible to be authors in this study were included as authors.

### Conflict of interest statement

The authors declare that the research was conducted in the absence of any commercial or financial relationships that could be construed as a potential conflict of interest.
